# The effect of toxic leadership on workplace deviance: the mediating effect of emotional exhaustion, and the moderating effect of organizational cynicism

**DOI:** 10.1186/s12912-024-02308-x

**Published:** 2024-09-19

**Authors:** Asmaa Kamal Ahmed, Mohamed Hussein Ramadan Atta, Ahmed Hashem El-Monshed, Alia Ibrahim Mohamed

**Affiliations:** 1 Faculty of Nursing, Fayoum University, Fayoum City, Egypt; 2 Psychiatric and mental- health nursing Department, Faculty of Nursing, Alexandria University, 9 Edmond Vermont Street - Smouha, Alexandria City, Egypt; 3 Department of Nursing, College of Health and Sport Sciences, University of Bahrain, Manama City, Bahrain; 4 Department of Psychiatric and Mental Health Nursing, Faculty of Nursing, Mansoura University, Mansoura, Egypt; 5 Faculty of Nursing, Zagazig University, Zagazig City, Egypt

**Keywords:** Toxic leadership, Workplace deviance, Emotional exhaustion, Organizational cynicism, And Staff nurses

## Abstract

**Background:**

Toxic leadership is abusive, destructive, and detrimental to nurses, can decrease nurse morale, increase stress levels, diminish organizational performance, and cause employee emotional exhaustion. Emotional exhaustion, a key component of burnout, has been linked to decreased job performance and increased likelihood of engaging in deviant behaviors at work. Organizational cynicism, as a negative attitude or distrust towards the organization and its leadership, may exacerbate the relationship between toxic leadership and workplace deviance. This study aims to explore how toxic leadership, workplace deviance, emotional exhaustion, and organizational cynicism are interrelated in the nursing profession.

**Method:**

A multicenter descriptive, cross-sectional research design was conducted at three university hospitals affiliated with three Egyptian governorates: Zagazig, El-Fayoum, and Alexandria. Two hundred forty-three nurses completed the Personal and Job-related Data Questionnaire, Toxic Leadership Scale, Emotional Exhaustion Scale, Workplace Deviance Scale, and Organizational Cynicism Scale. Mediation analysis using SPSS PROCESS revealed that emotional exhaustion indirectly influences the relationship between toxic leadership and workplace deviance, while hierarchical multiple regression showed that organizational cynicism moderates this relationship, with linear regression confirming the predictive impact of these variables on workplace deviance.

**Results:**

Toxic leadership exhibits a substantial positive influence on workplace deviance (B = 16.132, *p* < 0.001), as does emotional exhaustion (B = 8.760, *p* < 0.001) and organizational cynicism (B = 5.376, *p* = 0.036). Furthermore, the interaction terms of toxic leadership, organizational cynicism, and emotional exhaustion are significant (B = -3.012, *p* = 0.036). The negative coefficient of the interaction term implies a mitigating impact, indicating that the combined presence of high toxic leadership, high emotional exhaustion, and high organizational cynicism may counterintuitively reduce workplace deviance (t = -2.110).

**Conclusion:**

These results suggest that while toxic leadership, emotional exhaustion, and organizational cynicism each independently increase workplace deviance, their combined presence may unexpectedly reduce deviant behaviors.

**Implication for nursing:**

These results highlight the significance of tackling toxic leadership practices and cultivating a positive organizational culture to enhance a healthier work environment and reduce instances of workplace deviance. Healthcare settings should prioritize interventions to improve leadership practices, enhance employee well-being, and cultivate a supportive organizational climate.

**Supplementary Information:**

The online version contains supplementary material available at 10.1186/s12912-024-02308-x.

## Introduction

Effective leadership within the nursing profession has significantly impacted nurses’ attitudes, emotional well-being, and the prevention of workplace deviance behaviors. As Alsadaan et al. (2023) posited, a positive and supportive leadership environment instills a sense of value and motivation among nurses and influences their overall mindset and job satisfaction. Healthy leadership has emerged as a potent catalyst, delineating clear expectations, fostering open communication, and adeptly addressing conflicts within the workplace milieu [1]. Leaders exemplifying ethical conduct and cultivating a culture of respect and collaboration markedly diminish the likelihood of disruptive behaviors among nursing staff, fostering a positive and industrious work environment [2].

Despite the burgeoning discourse on toxic leadership within nursing settings, which can yield detrimental effects on both the work environment and nurses’ well-being, as expounded by Ofei et al. (2023), it is imperative to recognize the adverse consequences of such leadership styles. Toxic leaders often exhibit pernicious traits, including abusive communication, lack of empathy, and a proclivity for micromanagement [3]. The toxic work environment is characterized by pervasive negativity, hostility, and dysfunction, adversely affecting individuals’ well-being and organizational productivity [4].

Central to the concept of a toxic environment is toxic leadership, characterized by behaviors that significantly contribute to overall toxicity. Intemperate behavior, exemplified by leaders displaying anger, impatience, and unpredictability, creates an unsettling atmosphere, fostering stress and anxiety among team members. Narcissistic behavior, prioritizing personal needs over the collective welfare of the team, undermines collaboration and trust. Self-promoting behavior, emphasizing personal achievements at the expense of recognizing others’ contributions, fosters resentment and diminishes team morale. Additionally, humiliating behavior, such as belittling or publicly criticizing subordinates, damages self-esteem and corrodes a healthy work culture [4–6]. In a different light, these toxic leadership components contribute to an environment where employees feel disempowered, demotivated, and reluctant to voice concerns, perpetuating a cycle of negativity and hindering organizational success [3, 7].

Against the backdrop of toxic leadership, workplace deviance emerges as a nuanced construct, denoting intentional, counterproductive behaviors violating organizational norms and undermining workplace functionality [8]. Bennett and Robinson’s (2000) conceptualization categorizes workplace deviance into organizational and interpersonal dimensions. Organizational deviance involves actions directly harming the organization, such as theft and fraud, while interpersonal deviance encompasses behaviors like gossiping and interpersonal aggression targeting colleagues [9, 23]. This comprehensive framework facilitates understanding the multifaceted ways deviant behaviors manifest in the workplace [8].

Examining workplace deviance’s immediate and long-term effects on nurses’ emotional factors, particularly emotional exhaustion, is pivotal. Constant exposure to deviant behaviors contributes to heightened stress levels and emotional exhaustion among nurses [10]. The strain resulting from interpersonal conflicts, gossip, or theft depletes nurses emotionally, leading to burnout and reduced job satisfaction. This emotional exhaustion jeopardizes both individual nurse’s well-being and compromises the quality of patient care. Addressing workplace deviance becomes imperative for promoting a healthier work environment, safeguarding nurses’ emotional health, and enhancing healthcare team effectiveness [11, 12].

Organizational cynicism, a negative attitude or skepticism towards the organization, can result from perceived hypocrisy, injustice, or a lack of trust in leadership [13]. In nursing, organizational cynicism linked to toxic leadership, workplace deviance, and emotional exhaustion can have significant repercussions. Toxic leadership behaviors fostering cynicism can intensify when nurses perceive leaders as manipulative or insincere. This cynicism contributes to workplace deviance as nurses may engage in counterproductive behaviors in response to their disillusionment with the organization, eroding trust and creating a toxic work environment. Organizational cynicism is also associated with emotional exhaustion among nurses, leading to increased stress and reduced job satisfaction [14].

Recognizing the interconnectedness of organizational cynicism, toxic leadership, workplace deviance, and emotional exhaustion is characterized by a nuanced amalgamation of established models within organizational behavior and leadership studies [15]. This intricate interplay among toxic leadership, workplace deviance, emotional exhaustion, and organizational cynicism warrants thorough investigation to understand its implications for nurses’ well-being and organizational outcomes.

The primary objective of this study was to investigate the correlation between toxic leadership and workplace deviance among nursing professionals. Specifically, the study aimed to analyze how emotional exhaustion mediates this relationship. Additionally, the study sought to explore how organizational cynicism moderates this relationship. By elucidating these mechanisms, the study aimed to provide insights into the complex interplay between leadership, individual well-being, and organizational outcomes within nursing contexts.

### Literature review and hypothesis development

Toxic leadership within nursing settings is a pervasive challenge, characterized by behaviors such as abusive communication, lack of empathy, and micromanagement. This form of leadership fosters a toxic work environment, resulting in reduced job satisfaction, elevated turnover intentions, and increased instances of workplace deviance among nurses. Existing literature has offered valuable insights into the adverse impacts of toxic leadership on workplace outcomes within nursing contexts. Studies by Abd El-Aziz and Elsaiad (2021) and Ofei et al. (2023) have highlighted the correlation between toxic leadership and various adverse outcomes, including nursing absenteeism, deviant workplace behaviors, and increased turnover intentions [3, 16].

Furthermore, Rizani and colleagues (2022) studied how toxic leadership influences organizational performance through the deviant behavior of 100 employees. Using a quantitative approach, the study surveyed 100 respondents and found that toxic leadership significantly impacts organizational performance and employee deviant behavior. Additionally, the study revealed that employee deviant behavior mediates the relationship between toxic leadership and organizational performance, indicating a complex interaction between leadership and workplace outcomes. This underscores the importance of further exploring the mechanisms that drive the relationship between toxic leadership and deviant behavior in the workplace [17].

*H1: There is a significant positive relationship between toxic leadership and workplace deviance.*

Emotional exhaustion emerges as a critical mediator in this relationship. Emotional exhaustion refers to feelings of being emotionally drained and depleted as a result of chronic workplace stressors, which are often exacerbated by toxic leadership behaviors [11, 18]. Nurses experiencing emotional exhaustion may be more likely to engage in deviant behaviors as a means of coping with stress and asserting control in their work environment.

*H2: Emotional exhaustion mediates the relationship between toxic leadership and workplace deviance*,* implying that increased emotional exhaustion*,* at least partially*,* explains the impact of toxic leadership on deviant behaviors* [11, 18].

Moreover, the relationship between toxic leadership and workplace deviance is moderated by organizational cynicism. Organizational cynicism involves negative attitudes and perceptions toward the organization, typically arising from perceived injustices or distrust in leadership [14]. Elevated levels of organizational cynicism can exacerbate the negative impact of toxic leadership on workplace deviance. This occurs because nurses may become disillusioned and disengaged from the organization, which can result in increased occurrences of deviant behaviors.

*H3: Organizational cynicism moderates the relationship between toxic leadership and workplace deviance*,* indicating that the strength of this relationship varies based on the level of organizational cynicism* [14].

Additionally, the combination of high toxic leadership, high emotional exhaustion, and high organizational cynicism may result in the highest levels of workplace deviance, suggesting an interactive effect of these factors.

*H4: The combination of high toxic leadership*,* high emotional exhaustion*,* and high organizational cynicism will result in the highest levels of workplace deviance*,* suggesting an interactive effect of these factors.*

Finally, even after accounting for emotional exhaustion and organizational cynicism, the direct relationship between toxic leadership and workplace deviance may remain significant, indicating the unique contributions of each factor.

*H5: After accounting for emotional exhaustion and organizational cynicism*,* the direct relationship between toxic leadership and workplace deviance will remain significant*,* indicating the unique contributions of each factor.*

**Fig. 1:**
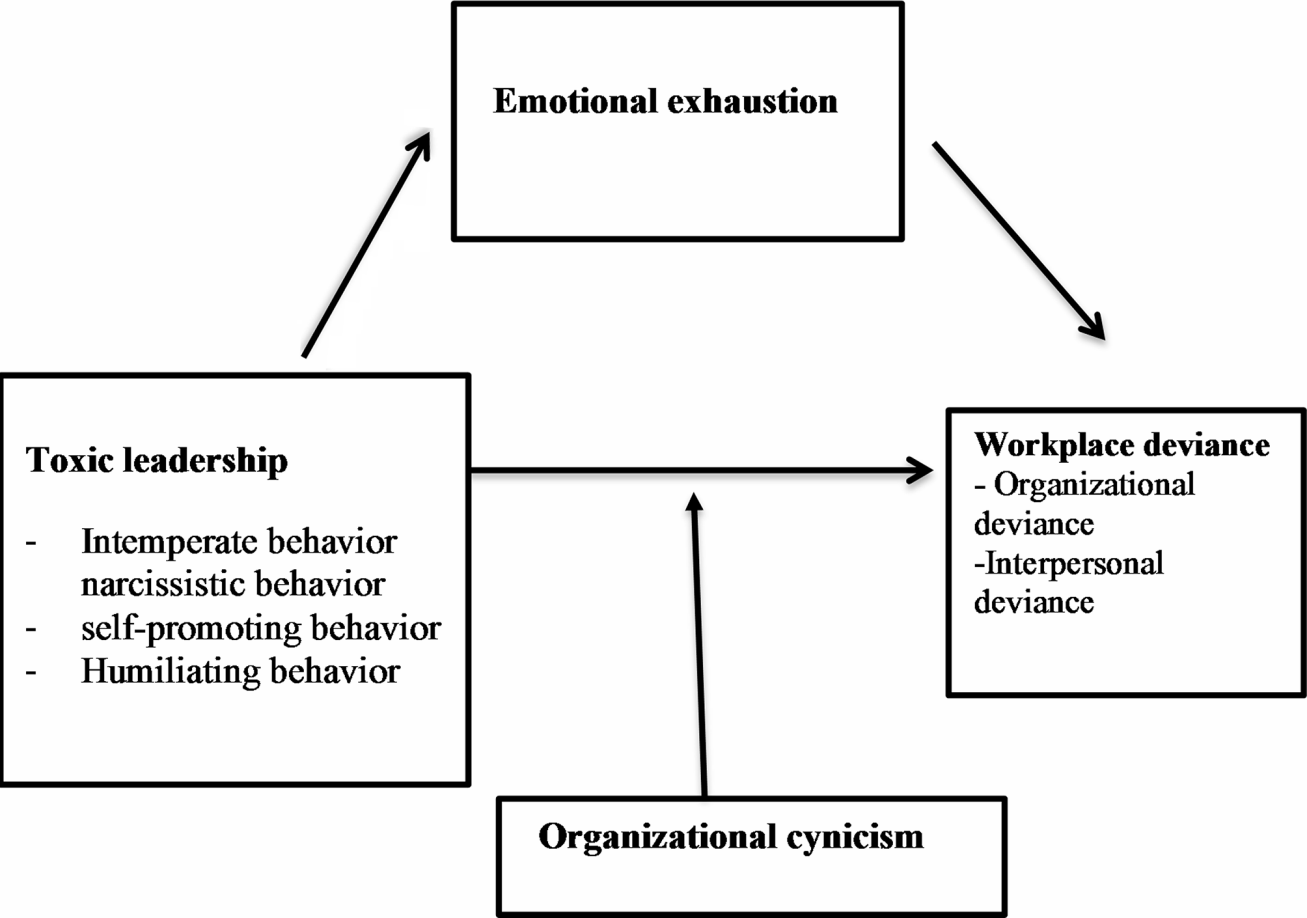
Toxic model

The intricate correlation among toxic leadership in Fig. (1), workplace deviance, emotional exhaustion, and organizational cynicism within the nursing context warrants thorough exploration, as this research delves into the psychological well-being of nurses. Insights into how toxic leadership dynamics impact mental health provide a nuanced understanding of the link between workplace variables and nurses’ behaviors, such as deviance. Recognizing the repercussions of toxic leadership on overall workplace culture and morale underscores the imperative of fostering positive environments.

The findings not only elucidate the interconnectedness of organizational cynicism, toxic leadership, workplace deviance, and emotional exhaustion but also offer valuable perspectives on how these factors collectively affect the quality of patient care. This research is a pivotal contributor to the field, paving the way for tailored intervention strategies, leadership training initiatives, and organizational adjustments to cultivate a healthier and more supportive work atmosphere.

## Method

### Research design

A multicenter descriptive, cross-sectional research study design was used for this study adhering to STROBE guidelines.

### Study setting

The study was conducted at three university hospitals affiliated with three big Egyptian governorates: Zagazig, El-Fayoum, and Alexandria.

### Population and sample size calculation

The population size for this study encompasses nurses employed in the previously mentioned study setting. These hospitals were selected due to their significance within the healthcare system and their diverse geographical locations, providing a broad representation of the nursing workforce in Egypt. To ensure a focused and relevant sample, inclusion criteria were set for participants in this study. Nurses from various departments and experience levels were selected to capture diverse perspectives, aiming for a comprehensive understanding of the phenomenon under investigation. In addition, we included those currently employed full-time or part-time at the selected university hospitals, regardless of specialization or tenure, with a minimum of six months of work experience to ensure familiarity with the organizational context and leadership dynamics. These criteria ensured that the study reflected insights from nurses actively involved in the healthcare environment with sufficient experience to contribute meaningful data. Nurses with less than six months of experience and those who declined to participate were excluded from the study.

In this study, the participants were nurses working in university healthcare institutions in three governorates. The sample size was calculated using an online sample size calculator at www.raosoft.com, based on an estimated maximum population of 10,000 nurses across the three governorates. With a 95% confidence interval and a margin of error of ≤ 5%, the required sample size was 230 nurses. Quota sampling was employed, with quotas allocated proportionally based on the population of each governorate using the latest statistics from the hospital Statistics. To achieve the desired sample size, it was decided in advance to invite 243 nurses to participate in the study, a decision influenced by previous research conducted among nurses and other healthcare providers.

### Tools of data collection

**Tool I** was regarding the respondent’s Personal and Job-related Data Questionnaire, e.g., age, working experience, educational levels, housing, marital status, and department (supplement 1).

**Tool II: Toxic Leadership Scale (ToxBH-N)**:

Labrague et al. (2020) developed the Toxic Leadership Scale to evaluate nurses’ experiences with toxic leadership [19]. It comprises 30 items across four sub-dimensions: intemperate behavior (15 items), narcissistic behavior (9 items), self-promoting behavior (3 items), and humiliating behavior (3 items). Participants rated each item on a Likert scale from 1 (not at all) to 5 (all the time). Scores on the ToxBH-NM scale were categorized as non-toxic (1.0–2.2 points), moderately toxic (2.3–3.6 points), and highly toxic (3.7–5.0 points). Higher composite scores on each sub-scale indicate a higher prevalence of toxic leadership behavior [19].

The scale demonstrated strong internal consistency, with an overall Cronbach’s α coefficient of 0.975. The Cronbach’s α coefficients for the four factors ranged from 0.895 to 0.965, indicating high reliability within each factor. The test-retest reliability of the ToxBH-NM Scale was 0.801, suggesting good stability over time. Reliability coefficients for the four factors ranged from 0.745 to 0.911, indicating consistent measurement across different administrations. The ToxBH-NM scale has shown validity and reliability among nurses in various studies [19, 20]. This study assessed the scale’s reliability using Cronbach’s alpha, yielding a value of 0.88.

**Tool III: Emotional Exhaustion Scale**:

The scale for emotional exhaustion utilized in this study was derived from the Maslach Burnout Inventory (MBI), created by Maslach and Jackson (1986) [21]. It comprises nine items (e.g., I feel emotionally drained from my work), each of the nine items rated on a five-point Likert scale ranging from 0 (never) to 5 (every day). The Emotional Exhaustion scale has shown validity and reliability among nurses, as evidenced by studies conducted by Soares et al. (2023) and Ling et al. (2020) [22]. In the original validation study by Maslach and Jackson (1986), Cronbach’s alpha coefficients for the Emotional Exhaustion dimensions were reported as 0.90 each. However, in the current study, the alpha coefficient for the total scale was 0.76 [21]. An official to use Emotional Exhaustion Scale from Mind Garden, Inc.: Order #69,235.

**Tool IV: Workplace deviance Sale** was developed by Bennett and Robinson (2000). The scale included 25 items and was divided into two subscales: organizational deviance and interpersonal deviance. Organizational deviance was further categorized into production deviance (7 items) and property deviance (7 items), while interpersonal deviance comprised personal aggression (7 items) and political deviance (4 items). Nurses rated their responses on a five-point Likert Scale ranging from 5 to 1, indicating frequency from “daily” to “never.” The total score indicated nurses’ perceptions of deviant behaviors, with cutoff points distinguishing between serious workplace deviance (≥ 30%) and minor workplace deviance (< 30%) [23]. The Workplace Deviance Behavior scale has been validated for use among staff nurses, as demonstrated in studies by Palo and Chawla (2015) and Hany, Hassan, and Badran (2020) [24, 25]. The Cronbach alpha coefficient measured the reliability of this tool, which was 0.82.

**Tool V: Organizational Cynicism Scale**:

The scale was developed by Brandes (1997) to measure organizational cynicism among staff nurses. It consists of 16 items (e.g., policies at this hospital made by the administration cause more problems than they solve, and hospitals here generally do not care enough about the needs of their employees). Scoring system: The response for each item was scored on a 5-point Likert scale ranging from [5] Completely agree to [1] Completely disagree, where each statement has five options [26]. The Organizational Cynicism scale has been reliable among nurses, as evidenced by studies conducted by Aly et al. (2016) [14] and Çaylak Altuntaş (2017) [27]. The Cronbach alpha coefficient measured the reliability of this tool, and it was 0.90.

### Study procedures

The Arabic translation of the tools was done after permission from the original authors. An official Tool III Emotional Exhaustion Scale permission was obtained from Mind Garden, Inc.: Order #69,235. These tools underwent face and content validity assessment by a panel of experts consisting of six assistant professors and three professors from the academic nursing staff. All necessary adjustments were made based on their feedback. A pilot study was conducted to ensure the instruments’ clarity, understanding, and applicability and to estimate the time needed to complete each questionnaire sheet. The pilot study involved 14 staff nurses, constituting 10% of the sample. These nurses were excluded from the primary study sample, and any required modifications were made based on their feedback. Data collection occurred over one month, from August 2023 to December 2023. The researchers explained the study’s aim to each staff nurse individually or in group meetings, and each nurse completed the questionnaire under the researchers’ guidance and supervision. The estimated time to complete each questionnaire sheet was approximately 20–30 min.

### Ethical considerations

The study received approval from the Ethics Committee of the Faculty of Nursing, Zagazig University (ID/ Zu. Nur. REC#:0102 on 21/6/2023). Permission to conduct the study was also granted by the medical and nursing directors of the hospitals and the head nurses of various departments, following an explanation of the study’s objectives. Participants were informed of their voluntary participation in the research, and the cover letter ensured confidentiality. Consent was obtained through questionnaire completion.

### Statistical analysis

Data analysis was conducted using AMOS 24 and SPSS 29. Hypothesis testing involved path analysis using the SPSS macro named PROCESS [28]. Categorical variables were described using numbers and percentages, while continuous variables were presented as Mean ± Standard Deviation (SD) for parametric data. The Pearson correlation test was utilized to assess relationships between continuous parametric variables. Mediation analysis was conducted with the SPSS macro PROCESS to explore emotional exhaustion’s indirect effects on the relationship between toxic leadership and workplace deviance. For moderation analysis, hierarchical multiple regression was employed to investigate the moderating role of organizational cynicism in the relationship between toxic leadership and workplace deviance. Linear regression analysis was used to predict the dependent variable, workplace deviance, based on the independent variables, including toxic leadership, emotional exhaustion, and organizational cynicism. Statistical significance was determined at *p* < 0.05, with highly significant results reported at *p* < 0.001.

## Results

Table 1 demonstrates that the study included 243 participants, with more than half being below 30 (58.4%), and the majority were female, constituting 69.3% of the sample. When examining marital status, more participants were married (51.9%) than unmarried individuals (34.6%). Regarding nursing qualifications, the majority held qualifications from technical institutes (45.3%), followed closely by those with bachelor’s degrees (42.8%). Analyzing the distribution of participants based on years of experience and departments, a significant majority had less than ten years of experience (70.4%), and 65.3% are working in inpatient departments.

The data reveal notable associations between personal-job-related characteristics and the study variables. For example, younger nurses (< 30 years) reported significantly lower scores in toxic leadership, workplace deviance, and organizational cynicism compared to their older counterparts (*p* < 0.001). Similarly, female nurses exhibited higher levels of toxic leadership and organizational cynicism (*p* = 0.004, *p* = 0.012) than male nurses. Urban residents reported significantly higher scores in toxic leadership, workplace deviance, emotional exhaustion, and organizational cynicism compared to rural residents (*p* < 0.001). Married nurses also displayed significant variations in toxic leadership and workplace deviance (*p* < 0.001), with widowed/divorced nurses showing the highest scores in these variables.

Further analysis based on years of experience shows that nurses with less than ten years of experience reported higher levels of workplace deviance and emotional exhaustion than those with more than ten years of experience (*p* < 0.001). Departmental variations were also observed, with nurses in inpatient departments reporting significantly higher levels of toxic leadership and workplace deviance than those in critical care units (*p* = 0.008, *p* < 0.001). Lastly, significant differences were found across hospitals, with nurses from Alexandria reporting the highest levels of toxic leadership, workplace deviance, emotional exhaustion, and organizational cynicism compared to those from Zagazig and Fayoum (*p* < 0.001).

**Table 1:** Toxic leadership, workplace deviance, emotional exhaustion, and Organizational Cynicism among Study respondents and their variation with the personal-job related data (*N* = 243)

Personal-Job related data		*N* (%)	Toxic Leadership		Workplace Deviance		Emotional Exhaustion		Organizational Cynicism	
			M (SD)	Test of Sig. (*P*)	M (SD)	Test of Sig. (*P*)	M (SD)	Test of Sig. (*P*)	M (SD)	Test of Sig. (*P*)
Age	> 30	142 (58.4)	3 (0.9)	*t*=-4.650 (< 0.001)***	60.9 (30.5)	*t*=-6.035 (< 0.001)***	30.9 (7.8)	*t*=-1.632 (0.104)	53.5 (12.1)	*t*=-3.532 (< 0.001)***
	≥ 30	101 (41.6)	3.6 (0.8)		85.13 (31.2)		32.6 (8)		59	
Gender	Male	74 (30.5)	3.5 (0.9)	*t* = 2.882 (0.004)**	83.4 (30.5)	*t* = 4.000 (< 0.001)***	32.5 (7.2)	*t* = 1.174 (0.242)	58.8 (10.8)	*t* = 2.538 (0.012)*
	Female	169 (69.3)	4.1 (0.9)		65.5 (32.6)		31.2 (8.2)		54.5 (12.5)	
Residence	Urban	157 (64.6)	3.4 (0.9)	*t* = 3.627 (< 0.001)***	81.7 (32.1)	*t* = 7.642 (< 0.001)***	33 (7.9)	*t* = 3.686 (< 0.001)***	57.6 (12.6)	*t* = 3.147 (0.002)**
	Rural	86 (35.4)	3 (0.8)		51.3 (24.6)		29.1 (7.3)		52.5 (10.6)	
Marital Status	Married	126 (51.9)	3.4 (0.9)	*F* = 10.842 (< 0.001)***	72.9 (34.3)	*F* = 11.602 (< 0.001)***	31.3 (8.1)	*F* = 0.357 (0.700)	57.2 (11.4)	*F* = 6.996 (< 0.001)***
	Unmarried	84 (34.6)	2.9 (0.8)		60.1 (28.7)		31.7 (7.6)		52.1 (13.2)	
	Divorced/ Widowed	33 (13.6)	3.7 (0.7)		90.8 (27.6)		32.6 (8.1)		60.1 (10.3)	
Nursing Qualification	Diploma	19 (7.8)	3.5 (0.8)	*F* = 2.047 (0.108)	80.6 (26.4)	*F* = 2.716 (0.045)*	32.2 (6.2)	*F* = 2.434 (0.066)	57.4 (9.6)	*F* = 2.311 (0.077)
	Technical institute	110 (45.3)	3.3 (0.9)		72.1 (33.9)		32.6 (7.6)		56.2 (12.6)	
	Bachelor’s degree	104 (42.8)	3.1 (0.9)		65.9 (32.8)		30.1 (8.4)		54.2 (12.1)	
	Master’s degree	10 (4.1)	3.8 (0.4)		91.4 (25.7)		35.1 (7.1)		64.2 (9.9)	
Years of Experience	> 10	171 (70.4)	3.3 (0.9)	*t* = 1.724 (0.086)	75.57 (33)	*t* = 3.414 (< 0.001)***	32.9 (7.7)	*t* = 3.908 (< 0.001)***	56.7 (12.4)	*t* = 1.812 (0.071)
	≥ 10	72 (29.6)	3.1 (0.8)		60 (30.6)		28.6 (7.7)		53.6 (11.4)	
Department	Inpatients	160 (65.3)	3.4 (0.9)	*t* = 2.691 (0.008)**	78.7 (32.1)	*t* = 5.374 (< 0.001)***	31.7 (8)	*t* = 0.220 (0.826)	56.9 (12.5)	*t* = 1.950 (0.052)
	Critical care units	83 (34.2)	3 (1)		56 (29.5)		31.4 (7.8)		53.7 (11.3)	
Hospital	Zagazig	95 (39.1)	2.6 (0.8)	*F* = 74.201 (< 0.001)***	43.2 (17)	*F* = 217.133 (< 0.001)***	29 (8)	*F* = 17.191 (< 0.001)***	50 (11.5)	*F* = 42.508 (< 0.001)***
	Fayoum	55 (22.6)	3.1 (0.8)		64.7 (28.1)		30.2 (7.6)		52.6 (11.6)	
	Alexandria	93 (38.3)	4 (0.5)		102 (16.1)		35.1 (6.7)		63.6 (8.5)	

*p < 0.05; **p < 0.01; ***p < 0.001. M = Mean; SD = Standard Deviation; Test of Sig. = Test of Significance; t = Independent-Sample T test; F = One-way ANOVA test Nursing diploma (intermediate education),* nursing technical institute (upper-intermediate education)*,* bachelor’s degree in nursing (higher education)*,* master’s degree in nursing (post-graduate education).*

**Fig. 2:**
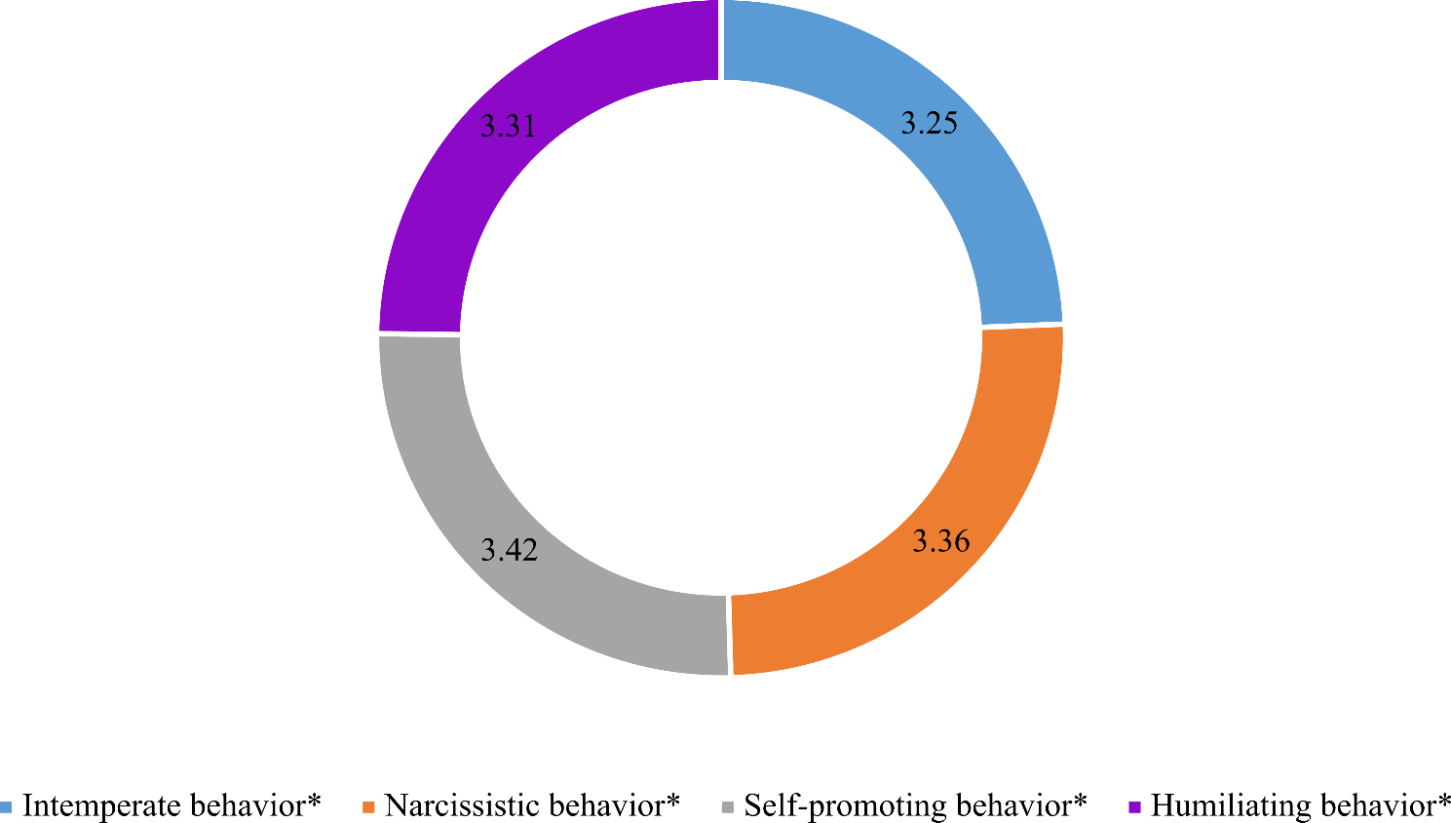
Composite means of toxic leadership behaviors (*N* = 243)

The study participants reported varying degrees of toxic leadership behaviors, as illustrated in Fig. 2. Self-promoting behavior emerged as the most prominently observed trait, with a mean score of 3.42 (SD = 1.13). Following closely, narcissistic behavior received a mean score of 3.36 (SD = 0.98). Humiliating behavior was reported with a mean score of 3.31 (SD = 0.94). In contrast, participants reported a comparatively lower mean score of 3.25 (SD = 1.01) for intemperate behavior, suggesting a somewhat lesser occurrence of this toxic leadership trait.

The bivariate correlation analysis supports Hypothesis 1 in Table 2, which posits a significant positive relationship between toxic leadership and workplace deviance. This table reveals that toxic leadership had a mean score of 3.31 (SD = 1.09). Workplace deviance had a mean score of 70.97 (SD = 33.02), while emotional exhaustion had a mean score of 31.65 (SD = 7.94), and organizational cynicism had a mean score of 55.84 (SD = 12.23). The correlation analysis revealed that toxic leadership was significantly and positively correlated with workplace deviance (*r* = 0.667, *p* < 0.001), emotional exhaustion (*r* = 0.532, *p* < 0.001), and organizational cynicism (*r* = 0.727, *p* < 0.001). Additionally, workplace deviance was significantly associated with emotional exhaustion (*r* = 0.527, *p* < 0.001) and organizational cynicism (*r* = 0.585, *p* < 0.001). Finally, emotional exhaustion was also strongly correlated with organizational cynicism (*r* = 0.622, *p* < 0.001). These findings suggest that higher levels of toxic leadership are closely linked with increased workplace deviance, emotional exhaustion, and organizational cynicism among nurses, highlighting the pervasive negative effects of toxic leadership on both individual and organizational outcomes.

**Table 2:** Means, standard deviations, and correlations of the study measures

Study Measures	Score^#^	(1)	(2)	(3)	(4)
Toxic Leadership (1)	3.31 (1.09)	1			
Workplace Deviance (2)	70.97 (33.02)	*r* = 0.667^***^	1		
Emotional Exhaustion (3)	31.65 (7.94)	*r* = 0.532^***^	*r* = 0.527^***^	1	
Organizational Cynicism (4)	55.84 (12.23)	*r* = 0.727^***^	*r* = 0.585^***^	*r* = 0.622^***^	1

*p < 0.05; **p < 0.01; ***p < 0.001.

^#^Scores are mean (SD) values

*r =* Pearson correlation

In Table 3, we present the results of the mediation analysis testing Hypothesis 2, which posits that emotional exhaustion mediates the relationship between toxic leadership (TL) and workplace deviance (WPD). The analysis reveals compelling evidence supporting this hypothesis. The direct effect of TL on WPD is significant (estimate = 0.6261, *p* < 0.0001), suggesting a robust influence of toxic leadership on workplace deviance. Furthermore, emotional exhaustion (EE) is a significant mediator in this relationship. The indirect effect of TL on WPD through EE is estimated at 0.1477 (Boot SE = 0.0411, Boot LLCI = 0.0719, Boot ULCI = 0.2313), demonstrating a substantial mediation effect. This result provides empirical support for the notion that the impact of toxic leadership on deviant behaviors is, at least partially, explained by the heightened levels of emotional exhaustion experienced by individuals within the organizational context. These findings contribute to a deeper understanding of the underlying mechanisms through which TL influences WPD, emphasizing the role of EE as a mediating factor. The observed effects underscore the importance of addressing EE in mitigating the negative consequences of TL behaviors in organizations.”

**Table 3:** Mediation analysis results for the relationship among toxic Leadership, emotional exhaustion, and Workplace Deviant

Paths	Estimate	SE	t	*p*-value	95% CI (LLCI, ULLCI)
TL → EE	0.1487	0.0158	9.7287	0.000	(0.1186, 0.1788)
TL → WPD	0.6261	0.0635	9.8563	0.000	(0.5009, 0.7512)
EE → WPD	0.9932	0.2272	4.3109	0.000	(0.5456, 1.4409)
Indirect effect of TL on WPD					
EE	0.1477	0.0411	--	--	(0.0719, 0.2313)

Notes: Confidence level for all confidence intervals in output: 95.0000, Number of bootstrap samples for percentile bootstrap confidence intervals: 5000, TL = Toxic Leadership, WPD = Workplace Deviant, EE = Emotional Exhaustion, SE = Standard Error, LLCI = Lower Limit Confidence Interval, ULCI = Upper Limit Confidence Interval

In support of Hypothesis 3, which posited that organizational cynicism moderates the relationship between toxic leadership and workplace deviance, our analysis revealed a significant moderation effect, as shown in Table 4. The interaction term, represented as ‘Moderator (TL*OC),’ demonstrated a positive and statistically significant coefficient (B = 0.015, *p* = 0.002), indicating that the strength of the relationship between toxic leadership (TL) and workplace deviance varies based on different levels of organizational cynicism (OC). The positive coefficient suggests that as the interaction term increases, the impact of toxic leadership on workplace deviance becomes more pronounced, providing empirical support for the moderating role of organizational cynicism.

While the main effects of Toxic Leadership (TL) and Organizational Cynicism (OC) individually were not significant (*p* = 0.384 and *p* = 0.094, respectively), the significant interaction term highlights that the impact of toxic leadership on workplace deviance varies depending on the level of organizational cynicism. The positive coefficient for the interaction term suggests that as organizational cynicism increases, the effect of toxic leadership on workplace deviance becomes more pronounced.

**Table 4:** The Moderation Effect of organizational cynicism on the relationship between toxic leadership and workplace deviance

Model		Unstandardized Coefficients		Standardized Coefficients	t	*p*-value	95% CI (LLCI, ULLCI)
		B	SE	Beta			
1	(Constant)	53.167	24.542	--	2.166	0.031	(4.819, 101.515)
	TL	-0.244	0.280	-0.211	-0.873	0.384	(-0.796, 0.307)
	OC	-0.797	0.475	-0.296	-1.680	0.094	(-1.733, 0.138)
	Moderator (TL*OC)	0.015	0.005	1.156	3.124	0.002	(0.006–0.024)

TL = Toxic Leadership, OC = organizational cynicism, SE standard error, LLCI = lower limit confidence interval, ULCI = upper limit confidence interval.

Dependent variable: Workplace deviance

In our investigation of the relationships among toxic leadership, organizational cynicism, emotional exhaustion, and workplace deviance, the regression analysis in Table 5 provides several key insights. The analysis was conducted to offer a comprehensive understanding of how these predictor variables interact simultaneously and contribute to workplace deviance, beyond the previous mediation and moderation analyses. The results indicate that toxic leadership has a substantial positive influence on workplace deviance (B = 16.132, *p* < 0.001), as do emotional exhaustion (B = 8.760, *p* < 0.001) and organizational cynicism (B = 5.376, *p* = 0.036). These findings are consistent with earlier analyses that highlighted the strong role of these variables.

Importantly, the interaction term of toxic leadership, organizational cynicism, and emotional exhaustion is significant (B = -3.012, *p* = 0.036). The negative coefficient of this interaction term suggests a nuanced effect, indicating that the combined presence of high toxic leadership, high emotional exhaustion, and high organizational cynicism may reduce workplace deviance in a counterintuitive way (t = -2.110). This finding underscores the complexity of the relationships among these factors and suggests that their combined effect may not be straightforward. Overall, these findings support Hypothesis 4 (H4), which posited that the combination of high toxic leadership, high emotional exhaustion, and high organizational cynicism would influence workplace deviance.

**Table 5:** Regression analysis of factors influencing Workplace Deviance: individual and interactive effects of toxic Leadership, Organizational Cynicism, and emotional exhaustion

Model		Unstandardized Coefficients		Standardized Coefficients	t	*p*-value	95% CI (LLCI, ULLCI)
		B	SE	Beta			
1	(Constant)	70.730	1.515	--	46.674	< 0.001	(67.745, 73.715)
	TL	16.132	2.239	0.488	7.204	< 0.001	(11.720, 20.543)
	OC	5.376	2.545	0.163	2.112	0.036	(0.362, 10.389)
	EE	8.760	2.186	0.266	4.007	< 0.001	(4.453, 13.068)
	Interaction (TL*OC*EE)	-3.012	1.427	-0.147	-2.110	0.036	(-5.824, -0.200)

TL = Toxic Leadership, OC = organizational cynicism, EE = Emotional Exhaustion, SE = standard error, LLCI = lower limit confidence interval, ULCI = upper limit confidence interval.

Dependent variable: Workplace deviance

## Discussion

Strong leadership is crucial for hospitals to survive and succeed in today’s competitive healthcare industry. Managers’ behavior directly affects their employees’ job satisfaction, mood, and performance levels. Positive leadership practices can create a supportive work environment, while toxic leaders can cause employees to feel alienated. Abusive management practices and insulting communication styles can undermine the values and norms of the institution, leading to inappropriate behaviors [29, 30]. Therefore, this study aims to fill this void by investigating how emotional exhaustion mediates and organizational cynicism moderates the relationship between toxic leadership and workplace deviance. This study aims to fill this void by investigating how emotional exhaustion mediates and organizational cynicism moderates the relationship between toxic leadership and workplace deviance.

### Relation between personal-job-related characteristics and the study variables

The study findings revealed that younger nurses reported significantly lower scores in toxic leadership, workplace deviance, and organizational cynicism than their older counterparts. This may be due to inexperienced nurses having more optimistic views of leadership and the workplace, as well as being less tolerant of toxic behaviors. However, their lack of experience might also shape their perceptions differently. Similarly, female nurses exhibited higher levels of toxic leadership and organizational cynicism than male nurses, potentially influenced by broader societal dynamics such as gender roles and workplace expectations, as women may be more sensitive to toxic behaviors stemming from their experiences in male-dominated fields.

Additionally, urban residents reported significantly higher scores in toxic leadership, workplace deviance, emotional exhaustion, and organizational cynicism compared to rural residents, which could be attributed to rural healthcare settings fostering more supportive workplaces that reduce instances of deviant behavior and cynicism. Furthermore, significant variations in toxic leadership and workplace deviance were observed among married nurses, with widowed or divorced nurses showing the highest scores in these areas. These differences suggest that the stressors faced by these groups vary, and the elevated scores among the widowed/divorced group indicate that personal trauma or loss can substantially influence workplace behavior and attitudes.

The study findings indicate that nurses with less than ten years of experience reported higher levels of workplace deviance and emotional exhaustion, which may be attributed to the challenges of adjusting to the rigorous demands of the profession and a lack of supportive systems early in their careers. There were also departmental variations, with nurses in inpatient departments experiencing significantly higher levels of toxic leadership and workplace deviance compared to those in critical care units. This discrepancy could be due to the influence of departmental culture and management styles on workplace dynamics; inpatient departments may have more hierarchical structures or insufficient leadership, creating an environment of toxicity and disengagement, whereas critical care units might promote collaboration and teamwork, leading to a healthier workplace [31]. Furthermore, notable differences across hospitals, particularly with Alexandria reporting the highest levels of toxic leadership and emotional exhaustion, suggest broader organizational issues such as management practices, staffing resources, and workplace policies that can exacerbate stress and cynicism among nurses.

This finding is consistent with a study by Abdallah and Mostafa (2021) in Egypt, which found a statistically significant positive correlation between total staff nurses’ perception of toxicity and their age, level of education, and years of experience [32]. Similarly, Singh, Dew, and Sengupta (2017) found that subordinate leaders’ apparent toxicity negatively correlates with age and education level [33].

### Relationship between toxic leadership and workplace deviance

The study findings provided empirical support for Hypothesis 1: Nurses’ perceptions of toxic leadership behaviors statistically significantly positively influence their attitudes and behaviors in the workplace, leading to an increase in workplace deviance. This could be attributed to toxic leadership behaviors, such as abusive supervision or a lack of support, creating a hostile work environment that fosters feelings of frustration, anger, and powerlessness among nurses. Our findings are consistent with Abd El-Aziz and Elsaiad (2021) conclusions, which demonstrate a statistically significant positive correlation between perceived toxic leadership and deviant workplace behaviors [16]. This aligns with the conclusions of Haider et al. (2018) in their study “Dark Side of Leadership: Employees’ Job Stress and Deviant Behaviors in the Pharmaceutical Industry,” where toxic leadership correlates with turnover intention and deviant behaviors significantly [31].

According to the current study’s results, the overall level of toxic leadership was moderate, consistent with previous research indicating a high percentage of toxic leadership dimensions. Studies have shown that toxic leadership behaviors among nurse managers directly impact nurses’ workload, leading to higher rates of adverse events such as verbal abuse, patient complaints, falls, infections, medication errors, and a decline in care standards. The evidence strongly suggests that addressing toxic leadership behaviors is essential for maintaining a safe and effective healthcare system. This finding is consistent with a study by Abdallah and Mostafa (2021) in Egypt, which found that staff nurses and their leaders exhibit high to moderate levels of toxic leadership [31]. Similarly, Ofei et al. (2022) found that registered nurses perceived the leadership behavior of nurse managers as toxic, with many managers displaying narcissistic leadership behavior [20].

Contrary to this, Mekawy and Ismail (2022) conducted a study in which staff nurses perceived minimal toxic leadership in their leaders [32]. Similarly, Abd El-Aziz and Elsaiad (2021) reported that slightly more than three-fifths of nurses perceived a low level of toxic leadership [16]. Furthermore, it was highlighted that staff nurses highly value leaders who facilitate positive relationships among team members, provide opportunities for training, address conflicts, and attentively address their concerns [33].

Our study found self-promoting behavior to be the most common toxic leadership trait among participants, followed by narcissistic and humiliating behavior. These behaviors can create toxic work environments, harm team morale and trust, and prioritize the leader’s success over the team’s well-being [34]. However, the study revealed a positive aspect of leadership, with a lower mean score for intemperate behavior, suggesting that leaders are less prone to outbursts of anger. Leaders must prioritize team members’ well-being and cultivate positive work environments for trust and collaboration. These results highlight toxic leadership traits in the workplace, which can negatively affect team performance and well-being. Organizations must address and mitigate these behaviors to foster a healthy and productive work environment [35].

This finding aligns with Abdallah and Mostafa’s (2021) study, which found that staff nurses perceived moderate self-interest and a lack of appreciation among their colleagues [32]. Similarly, Abou-Ramadan and Eid (2020) found that more than one-third of nursing staff believed their leaders displayed moderate levels of narcissism and unpredictable behavior [36]. However, our result differs from that of Mekawy and Ismail (2022), who found that only a quarter of nursing staff perceived their leader to exhibit highly intemperate and narcissistic behavior among toxic nurse managers [34].

### Emotional exhaustion mediates the relationship between toxic leadership and workplace deviance

Our study found that nurses’ emotional exhaustion is vital in connecting toxic leadership and workplace deviance, providing empirical support for hypothesis 2. This may be due to toxic leadership being a significant problem in healthcare organizations, leading to emotional exhaustion among nurses and increased workplace deviance. Our study found that behaviors like self-promotion, narcissism, and humiliation can create a hostile and stressful work environment, leading to burnout and decreased job satisfaction. This finding is consistent with the findings of Malik et al. (2019), who observed a similar relationship in their study involving employees. Additionally, emotional exhaustion can impair nurses’ ability to engage in effective decision-making and problem-solving, increasing the likelihood of deviant behaviors [37]. This finding is consistent with the research conducted by Mudallal, Othman, and Al Hassan (2017) [38]. Nurses who experience emotional exhaustion may be more inclined to engage in behaviors such as absenteeism, theft, or sabotage, as they may feel overwhelmed and find it challenging to cope with the demands of their work environment [36].

Moreover, emotional exhaustion can also lead to decreased organizational commitment, as nurses may feel disconnected from their work and less motivated to adhere to organizational norms and values (Opoku et al., 2021) [39]. This decreased commitment can further contribute to the likelihood of engaging in deviant behaviors, as nurses may feel less loyalty toward their organization and be more inclined to act in ways that serve their interests rather than those of the organization [37].

### Organizational cynicism moderates the relationship between toxic leadership and workplace deviance

According to the study, a positive coefficient shows that as the interaction between toxic leadership and organizational cynicism increases, the impact of toxic leadership on workplace deviance becomes more prominent. This finding supports hypothesis 3, which suggests that organizational cynicism moderates the relationship between toxic leadership and workplace deviance among nurses. The researchers believe that when nurses perceive their organization negatively, they are more likely to consider toxic leadership behaviors as reflective of the organization’s culture and values [40]. This can worsen the negative impact of toxic leadership, leading to a higher likelihood of deviant behaviors among nurses. Furthermore, organizational cynicism can amplify the effects of toxic leadership on nurses’ emotional exhaustion and job dissatisfaction, which can contribute to workplace deviance. However, a lack of organizational cynicism can act as a buffer against the adverse effects of toxic leadership [41].

This study highlights the crucial role of organizational cynicism in mediating the association between toxic leadership and deviant workplace behavior. These results are consistent with prior research by Hamzah (2023) and Chiaburu et al. (2013), which also emphasized the importance of organizational cynicism as a mediating variable. It is essential to explore additional factors that may impact this relationship. These studies underscore the significance of organizations addressing toxic leadership practices and fostering a positive work environment to reduce deviant behavior [42, 43].

### The combination of high toxic leadership, high emotional exhaustion, and high organizational cynicism will result in the highest levels of workplace deviance, suggesting an interactive effect of these factors

The study shows that toxic leadership, emotional exhaustion, and organizational cynicism can combine to reduce workplace deviance. However, statistical results do not imply causation, and the interpretation should be cautious. Nurses who are cynical about their organization may view toxic leadership as further evidence of dysfunction, leading to emotional exhaustion, disillusionment, and resentment. According to Elmaghraby, Hassan, and Elsetouhi (2023), toxic leadership directly relates to workplace deviance, even after considering emotional exhaustion and organizational cynicism. In summary, high levels of toxic leadership, emotional exhaustion, and organizational cynicism result in higher workplace deviance among nurses, indicating an interactive effect [44]. In addition, gaslighting and toxicity in the nursing workplace can lead to decreased job entrenchment [45].

### Limitation

A limitation of this study is its small sample size, which may not provide a comprehensive representation of the nursing population. Additionally, the study relies solely on questionnaires without incorporating direct observation, which could lead to potential biases and may not fully capture the complexities of the nurses’ experiences and workplace environments.

## Conclusions

The findings of this research significantly contribute to leadership literature and complement the study “The Effect of Toxic Leadership on Workplace Deviance: The Mediating Effect of Emotional Exhaustion and the Moderating Effect of Organizational Cynicism.” The study concludes that toxic leadership among nursing colleagues directly influences workplace deviance, with emotional exhaustion mediating this relationship. Organizational cynicism moderates the impact of toxic leadership on workplace deviance. Positive correlations were found between toxic leadership, workplace deviance, emotional exhaustion, and organizational cynicism.

### Implication for nursing practice policy

The findings underscore the detrimental impact of toxic leadership behaviors on organizational dynamics and employee behaviors. Organizations must recognize the potential risks associated with toxic leadership and take proactive measures to address and mitigate these behaviors.

Additionally, the results highlight the importance of managing employee well-being to minimize the negative consequences of toxic leadership. Implementing strategies to support employees in coping with emotional exhaustion may help reduce the likelihood of engaging in deviant behaviors in response to toxic leadership.

The moderating effect of organizational cynicism on the relationship between toxic leadership and workplace deviance emphasizes the significance of organizational culture in shaping employee responses to negative leadership behaviors. To mitigate the adverse effects of toxic leadership, organizations should focus on fostering a positive organizational climate and promoting trust and transparency.

Furthermore, the interactive effect of highly toxic leadership, high emotional exhaustion, and high organizational cynicism on workplace deviance highlight the importance of considering the combined impact of multiple factors in understanding employee behavior. Addressing these factors collectively may be more effective in managing and reducing workplace deviance.

Finally, toxic leadership behaviors are suggested to uniquely influence employee deviant behaviors, independent of their impact on emotional well-being and organizational perceptions. This underscores the need for targeted interventions to address toxic leadership behaviors directly.

## Electronic supplementary material

Below is the link to the electronic supplementary material.

Supplementary Material 1

## Data Availability

The datasets used and/or analyzed during the current study are available from the corresponding author on reasonable request.

## Abbreviations

ToxBH-N: Toxic Leadership Scale
TL: Toxic Leadership
OC: Organizational Cynicism
EE: Emotional Exhaustion
