# Supplementary material for: The effect of toxic leadership on workplace deviance: the mediating effect of emotional exhaustion, and the moderating effect of organizational cynicism
Source: BMC Nurs. 2024 Sep 19;23:669. doi: 10.1186/s12912-024-02308-x (PMC11414162; doi:10.1186/s12912-024-02308-x)
Supplement: Supplementary file 1 — Supplementary Material 1 [file 12912_2024_2308_MOESM1_ESM.docx]

**Personal and Job-related Data Questionnaire**

| **Personal-Job related data** | |
| --- | --- |
|  |  |
| **Age** | **> 30** |
|  | **≥ 30** |
| **Gender** | **Male** |
|  | **Female** |
| **Residence** | **Urban** |
|  | **Rural** |
| **Marital Status** | **Married** |
|  | **Unmarried** |
|  | **Divorced/**  **Widowed** |
| **Nursing Qualification** | **Diploma** |
|  | **Technical institute** |
|  | **Bachelor's degree** |
|  | **Master's degree** |
| **Years of Experience** | **> 10** |
|  | **≥ 10** |
| **Department** | **Inpatients** |
|  | **Critical care units** |
| **Hospital** | **Zagazig** |
|  | **Fayoum** |
|  | **Alexandria** |
